# Longitudinal association between smartphone ownership and depression among schoolchildren under COVID-19 pandemic

**DOI:** 10.1007/s00127-021-02196-5

**Published:** 2021-11-12

**Authors:** Masaki Adachi, Michio Takahashi, Hiroki Shinkawa, Hiroyuki Mori, Tomoko Nishimura, Kazuhiko Nakamura

**Affiliations:** 1 Department of Clinical Psychological Science, Graduate School of Health Sciences, Hirosaki University, 66-1 Hon-cho, Hirosaki-city, Aomori Japan; 2 Research Center for Child Mental Development, Graduate School of Medicine, Hirosaki University, Hirosaki, Japan; 3 Faculty of Education, Hirosaki University, Hirosaki, Japan; 4 Department of Neuropsychiatry, Graduate School of Medicine, Hirosaki University, Hirosaki, Japan; 5 Research Centre for Child Mental Development, Hamamatsu University School of Medicine, Hamamatsu, Japan

**Keywords:** Smartphone, COVID-19, Depression, Schoolchildren, Screen time

## Abstract

Under the COVID-19 pandemic, concerns regarding prolonged screen time and mental health effects in children have increased. We examined the association of depression with smartphone ownership in school children at four time points: September 2019, July 2020, December 2020, and March 2021. The analysis revealed an interaction between group and time, indicating that depressive symptoms among smartphone owners were significantly more severe than in the other group. These results were clearer for fourth-year students, pointing that smartphone possession at younger ages may be a risk factor for mental health in the new lifestyle caused by the COVID-19 pandemic.

**Supplementary Information:**

The online version contains supplementary material available at 10.1007/s00127-021-02196-5.

## Introduction

The COVID-19 pandemic affected the lives of children and adolescents everywhere around the world, as the public health precautions meant to reduce the infection rate included isolation policies, such as closing schools, parks, sports and recreational facilities, some of which already shown to have negative effects [1].A byproduct of these lifestyle changes is the increased screen time (ST) during the pandemic, as children and adolescents were forced to use electronic devices for communicational and educational purposes [2], even before COVID-19, it was well-known that prolonged sedentary behavior was linked to negative mental health outcomes in humans of all ages [3].

In recent years, the main cause of the rise of ST has been the continuously increasing usage of smartphones. The age when people begin using smartphones is continuously decreasing, though the concerns about the impact of ST on children’s development are scientifically confirm

ed. For instance, adolescents who excessively used smartphones had poor cognitive emotion

al regulation strategies [4], and experienced depression, anxiety, and daily dysfunctions [5]. Before COVID-19, adolescents used to spend around 6–8 h daily in sedentary positions (mostly ST [6]), and this behavior has presumably intensified during the pandemic [7].

Motivated by the concerns about the mental health effects of increased ST among children, some findings have already been revealed under the current pandemic. For example, a longitudinal study conducted in China between January 2020 and June 2020 showed that school closures and lockdowns may have increased problematic smartphone use and depression [8]. A study that examined the association between Internet use and psychological distress at two time points, in early November 2019 before the COVID-19 outbreak and at the end of March 2020 during the COVID-19 outbreak, reported that schoolchildren who increased their Internet-related activity by 15 or 30 min daily under the pandemic had increased levels of psychological distress [9]. Although there are several other studies that suggest an increase in Internet use and psychological distress under a pandemic [10–12], they only compare pre-COVID data with pandemic data or examine relatively short-term trends from the pre-COVID period to around June 2020; however, without follow-up for a longer span of time to reflect subsequent lifestyle changes, we investigated data showing yet unknown relationships between changes in mental health of Japanese schoolchildren at four time points (September 2019, July 2020, December 2020, and March 2021) starting from the pre-COVID-19 data, and possession of smartphones.

## Methods

### Study design and participants

The data for this study were partially augmented with data from the school survey of the Assessment of Preschool to Adolescence—Longitudinal Epidemiological (APPLE) study, a longitudinal study aimed at increasing knowledge of mental health trends and their confounders in a community sample in Japan [13]. Detailed information on Hirosaki City, Aomori Prefecture, where the survey was conducted, and the daily and cumulative number of COVID-19 cases in the Aomori Prefecture during the study period are shown in Online Supplement (Supplement Text 1 and Supplement Fig. 1).

The survey was conducted at each school in the city once a year until September 2019, and several times a year since 2020 with the aim of observing the impact of COVID-19 pandemic over time. From 2020 to 2021, three surveys were conducted: July 2020 (Wave 1), December 2020 (Wave 2), and March 2021 (Wave 3). These three surveys together with the one conducted in September 2019 (Wave 0), as a baseline, were included in this study. The involved children were in fourth to seventh grade (i.e., between the ages 9–12) at Wave 0. At each time point, we mailed letters containing information on the study to the parents or guardians of each child, and any lack of consent excluded the respective child from the study. The participating children were briefed about the purpose of the survey and completed the questionnaire in the classroom.

Questionnaires were distributed to 5204 children at each Wave and responses were received from 4875 children (boy: 50.1%) at all time points from Wave 0 to Wave 3 (Retention rate: 93.7%). Among the targeted children, 4227 guardians (boy: 49.8%) responded to whether they had a smartphone or not. (Response rate: 82.6%).

The protocol of the study was approved by the Committee on Medical Ethics of the Hirosaki University (IRB# 2015–055).

## Measurements

### Possession of smart phones

The possession of a smartphone was extracted from a set of questions used to infer socio-economic status in Japan [14]. Guardians were asked at one time point in Wave 1 about the exclusive usage of a smartphone by their children.

#### Indicators of mental health:

Depression was employed as indicator of mental health. Adolescents’ major depression, one of the most prevalent mental health problems in this age group, is associated with an increased risk for subsequent anxiety, mood, and even suicide. The Patient Health Questionnaire for Adolescents (PHQ-A) was used to screen for depression among children and adolescents, in a developmentally appropriate fashion, following Diagnostic and Statistical Manual (DSM-IV-TR) criteria [15]. It can be used as a severity measure, with scores ranging from 0 to 27 (high scores indicating more severe depression) and its Japanese version was developed and standardized in a general population sample of Japanese children and adolescents [16].

### Statistical analyses

#### Missing data

Evaluating the missing values of PHQ-A total score from Wave 0 to 3 with Little’s MCAR test, we found that there were missing not completely at random (*χ*^2^ = 144.92, df = 28, *p* < 0.001). Therefore, we performed the multiple imputation to complement the missing values [17]. The detailed procedure for missing value completion is shown in the Online Supplement (Supplementary Text 2).

#### Analytic plans

A linear mixed model was used for the analysis. The time variable provided the structure to the model with four waves of data (corresponding to Wave 0–Wave 3), and was entered as a fixed effect. Random intercepts and slopes were also entered, which allow different participants to have unique growth trajectories. Time invariant values of group (smartphone: own vs. not own) were entered into the model, as fixed effects, to predict changes in the time varying depression variable. The autoregressive covariance structure was chosen based on the best goodness-of-fit (as evaluated by the Akaike Information Criterion—AIC), compared to competing covariance structures. The restricted maximum likelihood-REML was chosen as the estimation method. Gender difference and economic impact of COVID-19, which were assumed to potentially influence the results, were added as covariates. As a supplementary analysis, a Chi square test was conducted to examine whether the percentage of those exceeding the cutoff value of PHQ-A at each time period (Wave 0–3) was significantly different between groups (smartphone own vs. not own).

## Results

A linear mixed model including the fixed effects of group (own vs. not own), time (Wave 0, Wave 1, Wave 2, Wave 3), and their interaction term as the predictors of depression was tested. Random intercepts and slopes were also included, which allowed each participant to have different growth trajectories. Results revealed a significant effect of group (*F* (1,4116) = 10.83, *p* = 0.001, *η* = 0.003), time (*F* (3,12,342) = 24.50, *p* < 0.001, *η* = 0.002) and interaction (*F* (3,12,348) = 6.79, *p* < 0.001, *η* = 0.001). Furthermore, the interaction of time and own group was significant at Wave 2 (*B* = 0.398, *SE* = 0.119, 95% CI [0.164, 0.632], *p* = 0.001) and Wave 3 (*B* = 0.492 *SE* = 0.119, 95%CI [0.258, 0.726], *p* < 0.001) compared with not own group at Wave 0, whereas the interaction at Time 1 was not significant (*B* = 0.189, *SE* = 0.119, 95%CI [− 0.045, 0.423], *p* = 0.113). Changes in depression scores between waves in each group are presented in Fig. S1. We continued with a linear mixed model conducted by grade level. The results showed a significant interaction only for the fourth grade (*F* (3,3189) = 3.07, *p* = 0.027, *η* = 0.003), indicating a different effect on depressive symptoms depending on whether the subjects owned or not smartphones. For the other grades, the interaction was not significant, although important differences were noticed between groups and times. The figures for the detailed statistical analysis and other basic statistics for this study are described in Online Supplement (Supplementary Text 3 and Supplementary Tables 1, 2, 3).

Chi square test group (smartphone own vs. not own) x cutoff value (above vs. below cutoff value) showed no significant difference in Wave 0 (*χ*^*2*^ = 0.130 *df* = 1, *p* = 0.728, *V* = 0.006), Wave 1 (*χ*^*2*^ = 3.197 *df* = 1, *p* = 0.081, *V* = 0.028) and Wave 2 (*χ*^*2*^ = 3.754 *df* = 1, *p* = 0.056, *V* = 0.030). While, there was significant difference in Wave 3 (*χ*^*2*^ = 4.826 *df* = 1,* p* = 0.030,* V* = 0.034). The results of the residual analysis showed that the own group had a significantly higher percentage above the cutoff (*p* < 0.05) and a significantly lower percentage below the cutoff (*p* < 0.05) compared to the expected value, while the not own group had a significantly lower percentage above the cutoff (*p* < 0.05) and a significantly higher percentage below the cutoff (*p* < 0.05). The detailed changes in the severity of depression for each group (smartphone own vs. not own) are shown in Online Supplement (Supplementary Table 4).

## Discussion

The results showed that referenced to Wave 0, which was not affected by the COVID-19 pandemic, the depression was higher in the smartphone own group compared to the not own group at Wave 2 and Wave 3. In addition, there was no difference in the percentage of depressive symptom scores that exceeded the cutoff value between smartphone owners and non-owners at Wave 0, but at Wave 3, the percentage of scores that exceeded the cutoff value was higher among owners and lower among non-owners. However, the mean total score of depressive symptoms did not increase among the smartphone owners, indicating that depressive symptoms did not worsen in the ownership group. Meanwhile, depressive symptoms in the non-owner group tended to improve from Wave 0 to Wave 3, suggesting that not having a smartphone may have a positive impact on depressive symptoms in the new lifestyle under the COVID-19 pandemic. Examining the results on grade level, these characteristics were particularly pronounced in the fourth grade, where the differences in depressive symptoms between the two groups, not present before COVID-19, became significant during the pandemic. This suggests that smartphone possession at a younger age may be a risk factor for mental health under the COVID-19 pandemic. However, we have not been able to examine whether the possession itself or other environmental factors that make smartphones available at an early age are more relevant risk factors. In previous studies, significant associations between children’s early exposure to smartphones and mothers’ smartphone addiction, and between mothers’ smartphone dependence and poor family functioning [18] were noted; hence, poor family functioning could also influence mental health deterioration in younger children.

To the best of our knowledge, no previous study has investigated yet the medium-term mental health transition under the COVID-19 pandemic. Other strengths of our investigation are its large scale and relatively high retention rate. However, it has some limitations, as it was conducted in a region of Japan, and it is not clear whether its results apply to other areas, taking also into consideration that the numbers of deaths and infections due to COVID-19 were lower in Japan than in other countries, and no strict lockdowns have been implemented (see Supplementary Text 1). The finding that depressive symptoms have not worsened in the entire sample may also be related to this condition in Japan. The differences noticed between smartphone owners and nonowners may partially be due to background factors rather than an effect of the smartphone use. Such background factors were not adequately controlled in this study. In addition, the effect size of the interaction obtained in this study is quite small and the results should not be overestimated. It is widely known that it is the “problematic use of smartphones” (e.g., problematic Internet use [7–9, 11, 12]) that affects mental health, although this study examined the transition of the effects of “smartphone possession and non-possession” on depression. The small effect size obtained may be related to the fact that the essential aspects of the question were not fully captured by simply asking whether or not the respondents had a smartphone.

## Supplementary Information

Below is the link to the electronic supplementary material.

Supplementary file1 (DOCX 91 KB)

## Data Availability

Not applicable.
