# Supplementary material for: Longitudinal association between smartphone ownership and depression among schoolchildren under COVID-19 pandemic
Source: Soc Psychiatry Psychiatr Epidemiol. 2021 Nov 12;57(2):239–43. doi: 10.1007/s00127-021-02196-5 (PMC8588933; doi:10.1007/s00127-021-02196-5)
Supplement: Supplementary file 1 — Supplementary file1 (DOCX 91 KB) [file 127_2021_2196_MOESM1_ESM.docx]

**ONLINE SUPPLEMENT APPENDIX**

**Supplementary Text 1.** Information about Hirosaki City

**Supplementary Figure 1.** Changes in infection status in the surveyed areas

**Supplementary Text 2.** Completion of missing values

**Supplementary Table 1.** Descriptive statistics for each wave of PHQ-A for all samples and for each grade

**Supplementary Text 3.** A linear mixed model conducted for each grade level

**Supplementary Table 2.** A linear mixed model with the depression score conducted for all sample

**Supplementary Table 3.** A linear mixed model with the depression score conducted for each grade level

**Supplementary Table 4.** Changes in severity of depression in each group (smartphone own vs. not own)

**Supplementary Table 5.** Descriptive statistics for each wave of PHQ-A for all samples and for each grade (before missing value completion)

**Supplementary Text 4.** A linear mixed model with the depression score conducted for all sample (before missing value completion)

**Supplementary Table 6.** A linear mixed model with the depression score conducted for all sample (before missing value completion)

**Supplementary Text 1.** Information about Hirosaki City

Hirosaki city is located in the northern part of Japan’s main island. This city is about 524.20 km^2^ in size, and the total population was reported as 177,411 in 71,152 households [1]. The taxable annual income per taxpayer in Hirosaki City is 2,687,000 Yen, which is close to the national level (2,747,000 Yen) [2]. Hirosaki City has 52 public elementary and junior high schools (35 elementary schools and 17 junior high schools), and there is only one private school (junior high school). About 99.4% of children are enrolled in public elementary and junior high schools.

Within the study period, there was no lockdown not only in Aomori Prefecture but also in Japan as a whole. The declaration of a “state of emergency issued” by the Japanese government is a request from the Prime Minister to the prefectural governors to take measures to protect the lives, health and livelihood of the people [3].　Specific measures such as refraining from going out or restricting events were decided upon, but they were based on self-restraint and did not restrict going out or travel between cities or other prefectures. This emergency declaration was issued in Aomori Prefecture only from April 16 to May 14, 2020, and has never been issued within the study period since then. Furthermore, as can be seen from Supplement Figure 1, the number of infected people in Aomori Prefecture during this period was very small, and the purpose of declaring a state of emergency in Aomori Prefecture during this period was mainly to prevent the spread of infection from other prefectures to Aomori Prefecture.

**References for Online Supplement.**

1. The Statistics Bureau of Japan. Population Census [Internet]. [cited 2021 June 4]. Available from: https://www.e-stat.go.jp/en/stat-search/files?page=1&layout=datalist&toukei=00200521&tstat=000001080615&cycle=0&tclass1=000001089055&tclass2=000001089057&tclass3=000001089059&stat_infid=000031473346&second2=1

2. Cabinet office: Population and economic data by municipality [Internet]. [cited 2021 June 4]. Available from: https://www5.cao.go.jp/keizai-shimon/kaigi/special/future/keizai-jinkou_data.html.

3. Cabinet Secretariat: COVID-19 Information and Resources [Internat]. [cited 2021 August 30]. Available from: https://corona.go.jp/en/

**
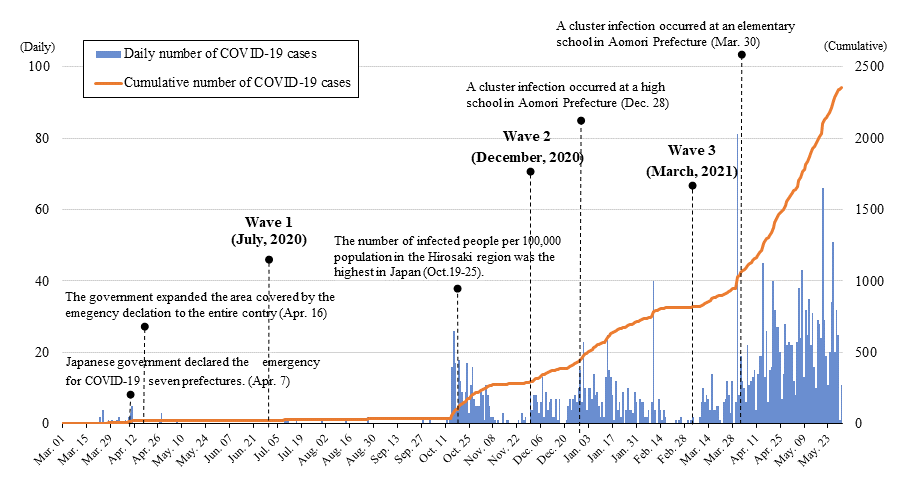
Supplementary Figure 1.** **Changes in infection status in the surveyed areas**

**Supplementary Text 2. Completion of missing values**

Among the four time points from Wave 0 to Wave 3, missing value completion based on the multiple imputation was performed for cases where PHQ-A total score data were available at half of the time points. Of the total sample of 5204 children, 4945 children responded at more than half of the time points (95.1%) and these samples were targeted for missing value completion. The percentage of missing values of PHQ-A in each wave was Wave 0 = 3.0%, Wave 1 = 2.1%, Wave 2 = 2.9%, and Wave 3 = 2.9%. We generated the imputation data using the missing value software of SPSS. Referring to Baraldi and Enders (2010), the number of imputation data generation was set to 20 [1]. Predictor variables included total PHQ-A scores from Wave 0 to Wave 3, smartphone ownership, grade, gender, and worsening economic conditions. The complete datasets (m = 20) were analyzed separately and the results are shown as an average of them. In order to be able to compare the data before and after completion, descriptive statistics and a linear mixed model results for data without missing value completion are shown in Online Supplement Text 4 and Supplement Table 5-6.

**References for Online Supplement.**

1. Baraldi AN, Enders CK. An introduction to modern missing data analyses. Journal of school psychology. 2010;48(1):5-37.

**Supplementary Table 1.** Descriptive statistics for each wave of PHQ-A for all samples and for each grade.

a) Upper section = Mean, Middle section = Standard deviation, Lower section = *n*

**Supplementary Text 3.** A linear mixed model conducted for each grade level

A linear mixed model including the fixed effects of group (own vs. not own), time (Wave 0, Wave 1, Wave 2, Wave 3), and their interaction term as the predictors of depression was tested. Random intercepts and slopes were also included, which allow each participant to have different growth trajectories.

4^th^ grade: Results revealed a significant effect of group *(F* (1,1063) = 5.41, *p* = .020, *η* = .003), time (*F* (3,3189) = 4.28, *p* = .005, *η* = .002) and interaction (*F* (3,3189) = 3.07, *p* = .027, *η* = .001). Furthermore, the interaction of time and own group was significant at Time 2 (*B* = .794, *SE* = .279, 95% CI [.246, 1.342], *p* = .005) and Time 3 (*B* = .635 *SE* = .279, 95%CI [.086, 1.183], *p* = .023) compared with not own group at Time 0, whereas the interaction at Time 1 was not significant (*B* = .388, *SE* = 0.279, 95%CI [-.159, .935], *p* = .165).

5^th^ grade: The results of a linear mixed model, the time factor was significant (*F* (3, 3300) = 19.67, *p* < .001, *η* = .002). While, group and interaction effects were not significant (group: *F* (1,1100) = 3.47, *p* = .063 *η* = .003, interaction: *F* (3, 3300) = 1.64, *p* =.178, *η* =. 001).

6^th^ grade: The results of a linear mixed model revealed group, time and interaction were not significant (group: *F* (1,934) = 3.81, *p* = .051, *η* = .004, time: *F* (3, 2802) = 0.77, *p* = .512, *η* = .002, interaction: *F* (3, 2802) = 1.29, *p* = .276, *η* =. 001).

7^th^ grade: The results of a linear mixed model, only the time factor was significant (group: *F* (1,1013) = .189, *p* = .664, *η* < .001, time: *F* (3, 3039) = 6.26, *p* < .001, *η* = .007, interaction: *F* (3, 3039) = 1.53, *p* =.205, *η* =. 001).

**Supplementary Table 2. A linear mixed model with the depression score conducted for all sample**

 Gender differences and the economic impact of the COVID-19 pandemic were added as covariates.

a) coefficient, b) Standard Error, c) CI = Confidence interval, d) Ref.: Reference category

**Supplementary Table 3.** A linear mixed model with the depression score conducted for each grade level

Gender differences and the economic impact of the COVID-19 pandemic were added as covariates.

a) coefficient, b) Standard Error, c) CI = Confidence interval, d) Ref.: Reference category

**Supplementary Table 4.** Changes in severity of depression in each group (smartphone own vs. not own)

PHQ-A: Patient Health Questionnaire modified for Adolescents

a) The severity classification and cut-off values were based on Kroenke (2001) [1].

**References for Online Supplement.**

1. Kroenke K, Spitzer RL, Williams JB. The PHQ-9: validity of a brief depression severity measure. J Gen Intern Med. 2001;16(9):606-13.

**Supplementary Table 5.** Descriptive statistics for each wave of PHQ-A for all samples and for each grade (before missing value completion)

a) Upper section = Mean, Middle section = Standard deviation, Lower section = *n*

**Supplementary Text 4.** A linear mixed model with the depression score conducted for all sample (before missing value completion)

A linear mixed model including the fixed effects of group (own vs. not own), time (Wave 0, Wave 1, Wave 2, Wave 3), and their interaction term as the predictors of depression was tested. Random intercepts and slopes were also included, which allow each participant to have different growth trajectories. Results revealed a significant effect of group (*F* (1,4106) = 10.80, *p* = .001, *η* = .003), time (*F* (3,11984) = 22.53, *p* <.001, *η* = .002) and interaction (*F* (3,12348) = 6.29, *p* <.001, *η* = .001). Furthermore, the interaction of time and own group was significant at Wave 2 (*B* = .397, *SE* = 0.123, 95% CI [.156, .637], *p* = .001) and Wave 3 (*B* = .482 *SE* = .123, 95% CI [.241, .723], *p* < .001) compared with not own group at Wave 0, whereas the interaction at Time 1 was not significant (*B* = .178, *SE* = 0.123, 95%CI [-.062, .418], *p* = .146).

**Supplementary Table 6.** A linear mixed model with the depression score conducted for all sample (before missing value completion)

Gender differences and the economic impact of the COVID-19 pandemic were added as covariates.

a) coefficient, b) Standard Error, c) CI = Confidence interval, d) Ref.: Reference category
